# Predicting the Failure Risk of Internal Fixation Devices in Chinese Patients Undergoing Spinal Internal Fixation Surgery: Development and Assessment of a New Predictive Nomogram

**DOI:** 10.1155/2021/8840107

**Published:** 2021-01-26

**Authors:** Chong Liu, Zide Zhang, Yuan Ma, Tuo Liang, Chaojie Yu, Zhaojun Lu, Guoyong Xu, Zequn Wang, Jiarui Chen, Jie Jiang, Tianyou Chen, Hao Li, Zhen Ye, Xinli Zhan

**Affiliations:** ^1^Guangxi Medical University, No. 22 Shuangyong Road, Nanning, Guangxi 530021, China; ^2^Spine and Osteopathy Ward, The First Affiliated Hospital of Guangxi Medical University, No. 6 Shuangyong Road, Nanning, Guangxi 530021, China; ^3^Beijing Haidian Hospital, No. 29 Zhongguancun Street, Haidian District, Beijing 100080, China

## Abstract

The current study is aimed at developing and validating a nomogram of the risk of failure of internal fixation devices in Chinese patients undergoing spinal internal fixation. We collected data from a total of 1139 patients admitted for spinal internal fixation surgery at the First Affiliated Hospital of Guangxi Medical University from May 2012 to February 2019. Of these, 1050 patients were included in the spinal internal fixation group and 89 patients in the spinal internal fixation device failure group. Patients were divided into training and validation tests. The risk assessment of the failure of the spinal internal fixation device used 14 characteristics. In the training test, the feature selection of the failure model of the spinal internal fixation device was optimized using the least absolute shrinkage and selection operator (LASSO) regression model. Based on the characteristics selected in the LASSO regression model, multivariate logistic regression analysis was used for constructing the model. Identification, calibration, and clinical usefulness of predictive models were assessed using C-index, calibration curve, and decision curve analysis. A validation test was used to validate the constructed model. In the training test, the risk prediction nomogram included gender, age, presence or absence of scoliosis, and unilateral or bilateral fixation. The model demonstrated moderate predictive power with a C-index of 0.722 (95% confidence interval: 0.644–0.800) and the area under the curve (AUC) of 0.722. Decision curve analysis depicted that the failure risk nomogram was clinically useful when the probability threshold for internal fixation device failure was 3%. The C-index of the validation test was 0.761. This novel nomogram of failure risk for spinal instrumentation includes gender, age, presence or absence of scoliosis, and unilateral or bilateral fixation. It can be used for evaluating the risk of instrumentation failure in patients undergoing spinal instrumentation surgery.

## 1. Introduction

The field of spine surgery has undergone tremendous changes for the past 100 years. The development of spinal instrumentation and fusion over the past three decades has brought the most significant advances [1]. The use of internal fixation systems in the spine is essential for stabilizing the structure of the spine, restoring its endurance, and protecting its function [2]. For the last ten years, the widely used instrument system in spinal surgery is the pedicle screw fixation. This was first used in the treatment of spinal fractures and later extended to spinal deformities, spinal tumors, lumbar spondylolisthesis, and low-back pain disorders [3, 4].

However, pedicle screw fixation has a probability of complications that require reoperation. Complications associated with internal fixation devices include screw and rod breakage, screw loosening, screw pullout, slippage of the connector, and loss of correction owing to implant failure. The most typical complication is screw loosening, with the reported incidence of 0.8%–27% and may even exceed 50% in patients with osteoporosis [4–6]. The causes of screw loosening are related to several factors, such as stress shielding or reduced load transferred through bone tissue, remodeling of bone around the screw, and bone microfracture owing to overload [7, 8]. Secondly, high strain at the bone-screw interface owing to insufficient anterior support may also lead to screw loosening [9]. Also, the presence of wear debris can cause osteolysis and the consequent risk of screw loosening [10]. Finally, the implant-related deep infection may also lead to screw loosening [6]. The failures are most commonly owing to the extension of the indications or innovative techniques. Surgery is the recommended treatment for thoracolumbar Magerl's A1 or A2 without neurological impairment. Surgery yielded the best results after six months, including spinal alignment, return to work, and reduced complications [11]. In an animal model, posterior medial costotransversectomy produced a significant deformity in minipigs. It requires detachment from the paravertebral musculature along the spinal contour, resulting in frank damage to the spine muscle-ligament structure (tension elements). However, with a posterior paramedian or video-assisted thoracoscopic anterior approach, this musculature remains intact, with the only tensional component injury occurring at the level of costotransverse joint ligaments [12]. Also, as spinal instrumentation implants are commonly located at key locations such as the spinal cord and near nerve roots, the fragile nature of such tissues determines that when internal fixation fails, their displacement and the consequences of secondary spinal instability can be harmful. It may lead to permanent neurological injury or death, with negative social impact and expenditure [13, 14].

Considering a large number of applications of spinal internal fixation systems and complications annually, factors affecting implant stability should be studied to largely avoid complications. Therefore, the current research was aimed at developing and validating a nomogram for predicting the failure of the spinal internal fixation system using common features.

## 2. Patients and Methods

### 2.1. Patients and Study Design

A retrospective study was conducted on the main patients who underwent spinal internal fixation surgery at the First Affiliated Hospital of Guangxi Medical University (Guangxi, China) from May 2012 to February 2019. Among them, 89 patients were included in the internal fixation failure group and 4262 patients were included in the normal spinal fixation group (1050 patients were randomly selected for this study).

The inclusion criteria for the spinal internal fixation failure group include

- Patients admitted to the hospital for internal fixation from May 2012 to February 2019 underwent surgery or revision surgery
- Posterior screw-rod internal fixation system: rod fracture, screw fracture, screw loosening, screw-rod connection point loosening, cross-rod loosening, screw pulling, transparent area (there is a low-density area with a width greater than 1 mm around the screw under X-ray), and double halo sign (there is also a bone edge area with both peripheral and peripheral radiation impermeable around the screw under X-ray) [15].
- Titanium cage and titanium plate internal fixation system appear titanium cage displacement, titanium plate loosening, and fixing screw loosening
- In the occipitocervical internal fixation system, screw loosening and plate displacement occur
- Patients with the loosening of upper and lower hook displacement, the disintegration of upper and lower hooks, breakage of Harrington rod, and broken wire in the Harrington rod internal fixation system

The exclusion criteria for the failed spine fixation group included

- Patients who did not meet the inclusion criteria
- Patients undergoing removal, adjustment, or revision surgery within one week after internal fixation
- Patients with an inconsistent diagnosis between intraoperative internal fixation failure and imaging diagnosis
- Patients with imperfect clinical and imaging data

The inclusion criteria for the normal group undergoing spinal instrumentation included

- Patients admitted for spinal internal fixation surgery from May 2012 to February 2019

The exclusion criteria for the normal group undergoing spinal instrumentation included

- Patients who did not meet the inclusion criteria
- Patients with spinal internal fixation failure
- Patients with imperfect clinical and imaging data

This study was reviewed and approved by the local institutional review board; informed consent was obtained from all patients.

### 2.2. Evaluation Variables

We collected the records of the primary diseases, age, gender, height, weight, occupation, and marital status of the patients using the His medical system at the First Affiliated Hospital of Guangxi Medical University. Height and weight were used to calculate the body mass index. The morbidity of underlying diseases includes the presence or absence of coronary heart disease, diabetes mellitus, and hypertension. Indicators collected using the picture archiving, and the communication imaging system for patients included the number of vertebral bodies crossed by internal fixation, number of vertebral bodies fixed by internal fixation, presence or absence of scoliosis and crossbars, and unilateral or bilateral fixation.

### 2.3. Training and Validation Tests

For the included experimental and control group cases, the *caret* package of *R* (Version 3.6.0: https://www.R-project.org/) was used for random selection and divided into training test and validation tests.

### 2.4. Statistical Analysis

All data were presented as counts (%). The *R* software was used for statistical analysis. The least absolute shrinkage and selection operator (LASSO) method is suitable for the reduction of high-dimensional data for selecting risk factors with the best predictive features from patients undergoing spinal internal fixation surgery. The features with nonzero coefficients in the LASSO regression model were selected [16, 17]. Then, based on the selected features of the model, a prediction model was constructed using multivariate logistic regression analysis. The features were considered as odds ratio having 95% confidence interval (CI) and as *P* value. The statistical significance levels were all two-sided. By introducing all the selected features and analyzing the statistical significance levels of the features, the statistically significant predictors and potential possible features were applied to develop a model of risk prediction for failure after internal spinal fixation. A calibration curve was constructed to evaluate the calibration of the failure nomogram of the spinal internal fixation device [18]. An important test statistic indicates that the model cannot be completely calibrated. For quantifying the discriminatory performance of the failure nomogram of spinal instrumentation devices, Harrell's C-index was measured [19]. For determining the clinical usefulness of a nomogram of spinal instrumentation failure, a decision curve analysis was performed by quantifying the net benefit at different threshold probabilities in the spinal instrumentation group [20]. The net benefit is calculated by subtracting the proportion of all false-positive patients from the proportion of truly positive patients and weighing the relative harm of abandoning intervention against the negative consequences of unnecessary intervention. A validation test was used to assess the readiness of the constructed failure risk nomogram.

## 3. Results

### 3.1. Patient Characteristics

**Figure 1:**
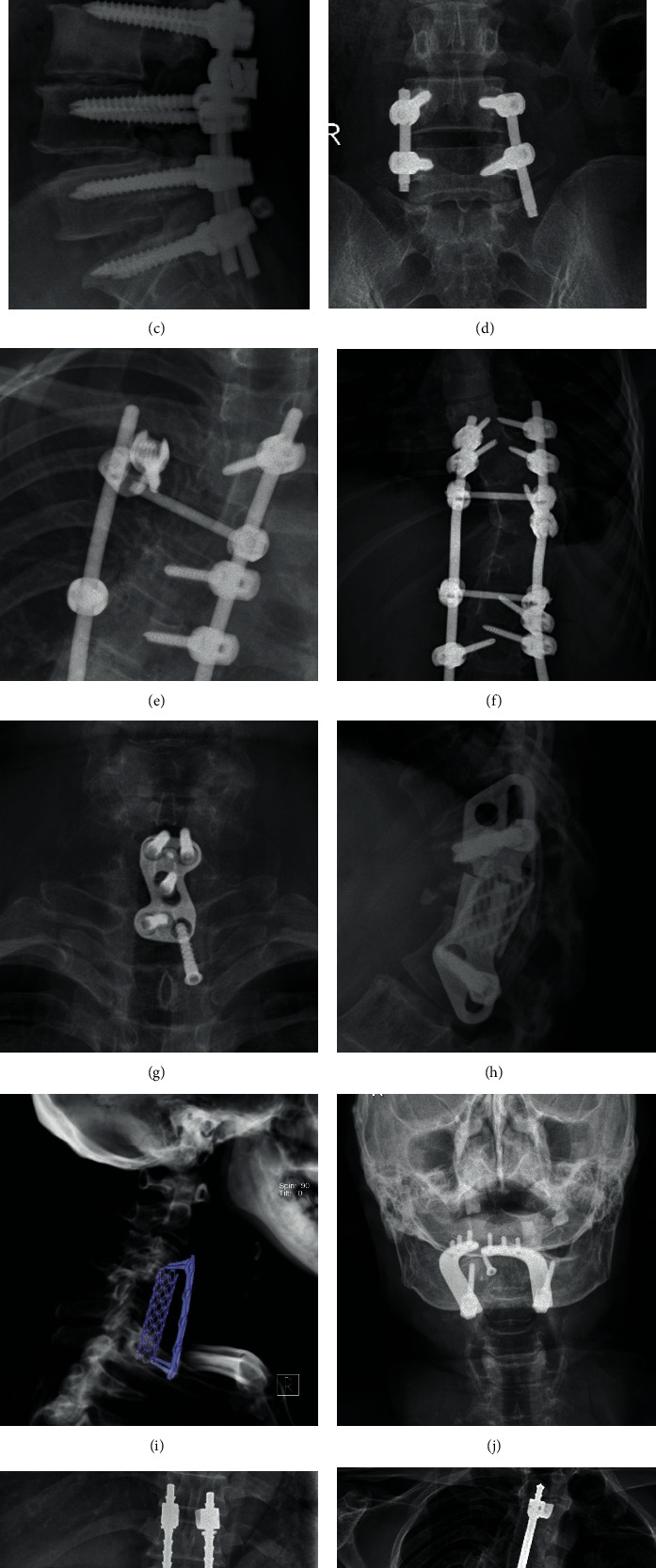
Typical cases of internal fixation failure: (a) screw fracture, (b) broken rod, (c) screw cap loosening, (d) screw rod connection loosening, (e) cross rod loosening, (f) pulling nail, (g) titanium plate nail loosening, (h) titanium plate loosening, (i) titanium cage loosening displacement, (j) neck pillow fusion nail loosening, (k) Harrington rod fracture, and (l) Harrington rod wire fracture.

A total of 89 patients were included in the internal fixation failure group and 4262 patients in the normal spinal fixation group (1050 patients were randomly selected for this study). Tables 1 and 2 present all data for patients in the training test and validation test, respectively. Typical cases of the internal fixation failure group were illustrated in Figure 1.

### 3.2. Feature Selection

**Figure 2:**
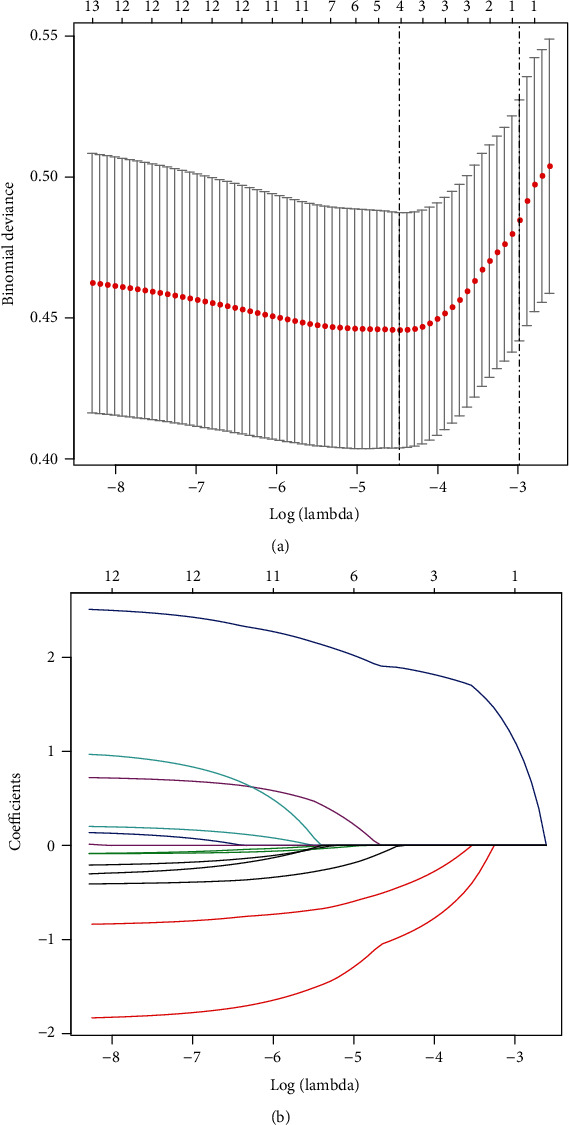
Feature selection using the LASSO binary logistic regression model. (a) Feature selection by the LASSO binary logistic regression model. By verifying the optimal parameter (lambda) in the LASSO model, the partial likelihood deviance (binomial deviance) curve was plotted versus log (lambda). Dotted vertical lines were drawn based on 1 SE of the minimum criteria (the 1-SE criteria). (b) Feature selection by the LASSO binary logistic regression model. A coefficient profile plot was produced against the log (lambda) sequence in Figure 2(a). Four features with nonzero coefficients were selected by optimal lambda. LASSO: least absolute shrinkage and selection operator; SE: standard error.

Based on the results of 799 patients in the training test (approximately 4 : 1 ratio, Figures 2(a) and 2(b)), 14 features were simplified to four features, and coefficients were nonzero in LASSO regression. These characteristics include gender, age, presence or absence of scoliosis, and unilateral or bilateral fixation.

### 3.3. Development of a Personalized Prediction Model

**Figure 3:**
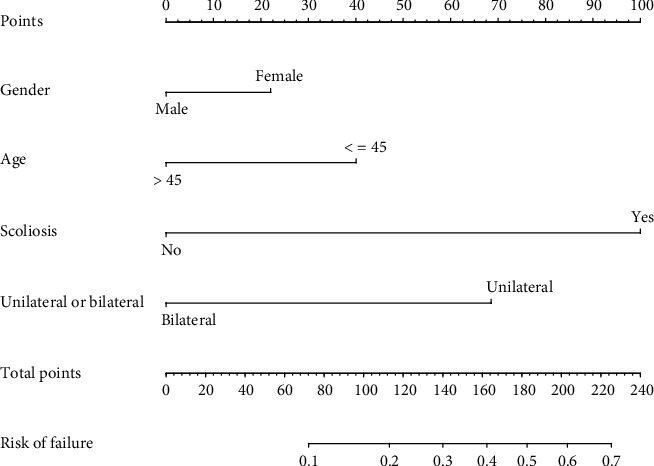
Construction of a nomogram for the failure of spinal internal fixation devices.

Gender, age, presence or absence of scoliosis, and unilateral or bilateral fixation of logistic container results are illustrated in Table 3. Following this, a model containing the above characteristics was developed and displayed as a nomogram (Figure 3).

### 3.4. Apparent Performance of the Risk Nomogram for Spinal Instrumentation Failure in the Cohort

**Figure 4:**
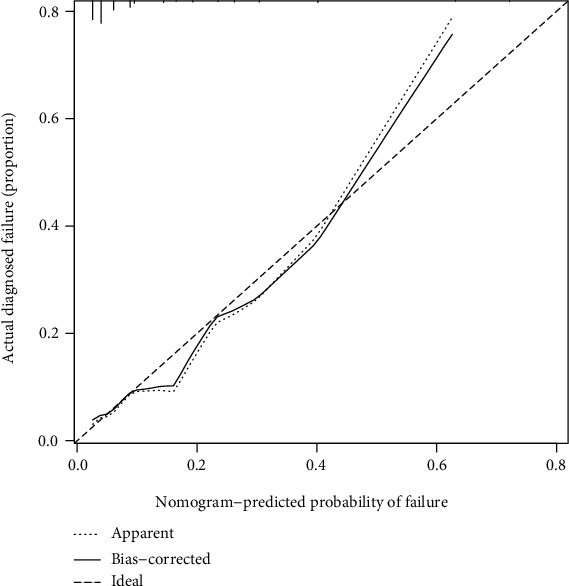
Calibration curves for predicting the risk profile of spinal instrumentation failure in the cohort. The *x*-axis represents the predicted risk of spinal instrumentation failure. The *y*-axis represents the actual diagnosis of spinal instrumentation failure. The diagonal dashed line represents an ideal perfect prediction model. Solid lines represent the performance of the nomogram, where closer proximity to diagonally dashed lines represents better prediction.

**Figure 5:**
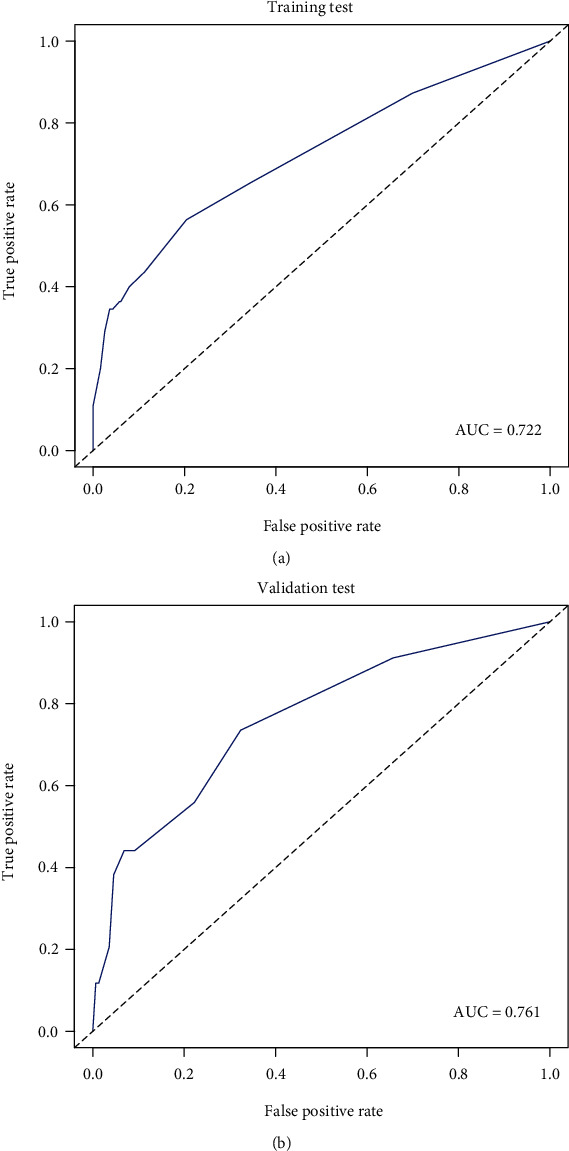
The AUC for training and validation tests. (a) Training test. (b) Validation test. AUC: area under curve.

For 799 patients in the cohort, predicting the risk of spinal instrumentation failure using nomogram calibration curves demonstrated moderate consistency (Figure 4). The C-index of the nomogram was 0.722 (95% CI: 0.644–0.800). The C-index of the validation test was 0.761 (95% CI: 0.675–0.847), indicating that the model had moderate predictive accuracy. The area under the curve (AUC) for training and validation tests were 0.722 and 0.761, respectively (Figure 5(a) and 5(b)). Collectively, the apparent performance demonstrated moderate predictive power in the nomogram of the risk of spinal instrumentation failure.

### 3.5. Clinical Use

**Figure 6:**
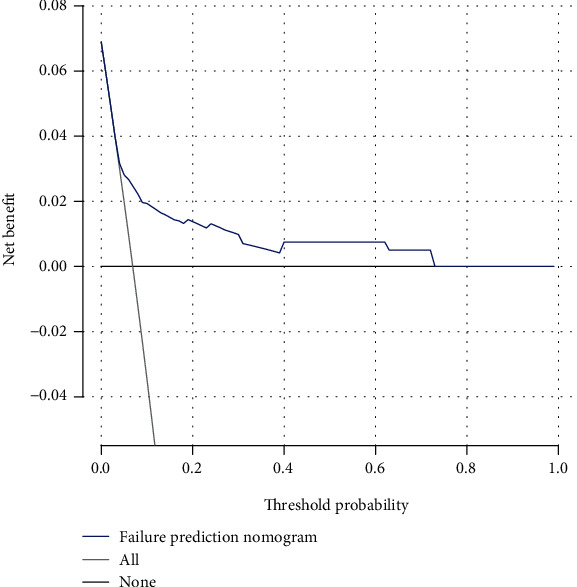
Decision curve analysis of failure risk nomogram of spinal internal fixation device. The decision curve showed that if the threshold probability of a patient and a doctor is >3% and<72%, respectively, using this failure risk nomogram in the current study to predict failure risk adds more benefit than the intervention-all-patients scheme or the intervention-none scheme.

The decision curve analysis of the risk nomogram for spinal instrumentation failure is illustrated in Figure 6. The decision curve demonstrated that when the threshold probabilities for a patient and physician is >3% and<72%, respectively, using this risk nomogram for predicting the risk of spinal instrumentation failure would be more beneficial than the existing schemes. Within this range, the net benefit was comparable with several overlaps, based on the risk nomogram.

## 4. Discussion

Nomograms are commonly used tools to estimate prognosis in oncology and medicine [21]. The current study is the first to apply the nomogram for assessing the failure rate of spinal internal fixation devices. We developed and validated a novel tool for predicting spinal instrumentation failure, using four readily available variables. By integrating demographics, reasons for initial surgery, and imaging indices into the nomogram, the prediction of spinal instrumentation failure is individualized. The current studies provide a relatively accurate predictive tool for the failure of spinal internal fixation surgery. The medium C-index value of the training and validation datasets identified that this nomogram could be widely and accurately used for its large sample size [22].

In risk factor analysis, failure of spinal internal fixation devices was associated with gender, age, presence or absence of scoliosis, and unilateral or bilateral fixation. The nomogram indicates that female, young age, scoliosis, and unilateral fixation may be the key factors in the risk of surgical failure of spinal instrumentation.

Osteoporosis is a major global health problem, with approximately 10 million people currently diagnosed with the disease, with approximately 82% of women [23, 24]. Osteoporosis reduces bone mass through negative bone remodeling, leading to the susceptibility of patients to spinal fracture, stenosis, and deformity; thus, surgical correction of these problems in such patients is challenging. Although pedicle screws are the most common tools for posterior fixation in the thoracic and lumbar spines, they are pulled out, loosened, and migrated owing to the risk of failure of the bone-screw interface in patients with osteoporosis [25, 26]. Therefore, female patients may be the main cause of spinal instrumentation failure due to the high incidence of osteoporosis. Previous studies have shown that higher spinal load may lead to implant subsidence, pedicle screw loosening, and even implant failure and may also be a cause of low back pain [27]. In China, patients aged below 45 years should engage in the necessary study and work after spinal internal fixation surgery, which may lead to increased load on the spine and cause the failure of spinal internal fixation devices.

Previous studies have demonstrated that 518 (4.2%) of 12,248 screws in adolescent scoliosis surgery were malpositioned. Also, 12 studies specifically addressed 11,928 pedicle screw implants with an incidence of 4.3% [28]. Akazawa et al. observed that the incidence of rod fracture was 5.2% in patients with spinal deformities who underwent spinal correction and fusion. Implant fracture is primarily the rod fracture, and the use of iliac screws and small diameter rod fractures is the common risk factors [29]. Also, Abul-Kasim et al. found that in 81 consecutive patients undergoing scoliosis surgery, one-third of patients demonstrated slight screw loosening after two years of interventional therapy [30]. Young scoliosis patients tend to have better bone quality than older patients. However, long spinal fixation corrections may impose greater mechanical forces and lead to screw loosening [31]. Nevertheless, unilateral fixation is more likely to lead to internal fixation device failure. Although unilateral fixation is a recommended treatment for lumbar spondylolisthesis, correct case selection is essential. The conditions such as obesity and osteoporosis should be prioritized and fixed bilaterally [32]. Aoki et al. found that degenerative scoliosis had a higher risk of cage displacement when unilateral pedicle screw fixation and bullet-shaped cages were used. Furthermore, when the posterior edge of the fusion cage was behind the posterior edge of the endplate, the cage was more likely to migrate after surgery [33]. Therefore, clinicians can predict the risk of internal fixation device failure according to the patient's situation, tailor a reasonable surgical program for patients, and formulate effective interventions for patients during the postoperative follow-up.

Risk prediction tools for spine instrumentation failures can be used through personalized risk prediction. Then, the clinicians can tailor reasonable and effective measures according to the results for reducing the risk of the internal fixation device failure in patients. We have developed an effective risk prediction tool for spinal instrumentation failure that can help clinicians in the early identification of high-risk patients. Moreover, it can serve as a user guide in clinical studies for the best patient selection for spinal internal fixation surgery. For example, the developed nomogram will guide researchers in selecting reliable patients with low risk by conducting clinical trials. When conducting retrospective studies, we can also eliminate some high-risk patients, thus making the analysis more reliable. Early interventions, such as antiosteoporosis therapy, avoidance of high-intensity exercise after surgery, and proper selection of unilateral and bilateral fixation, will benefit high-risk patients at the treatment initiation. Appropriate percutaneous vertebroplasty for the treatment of vertebral fractures caused by osteoporosis or tumor infiltration can significantly improve the pain and quality of life of patients [34]. In the pedicle screw systems, it is recommended that the rod should be placed on the screw head at a straight vertical angle, and the screw cup should be applied correctly to avoid mechanical failure. The use of variable angle (multiaxial) screws minimizes the need for rod contours, thereby avoiding prestressing loads applied to the structure and reducing early structural failure. When tightening the nut, exit the adjustment nut and rotate counterclockwise until the screw head loosens and produces a sound [35].

Therefore, an accurate prognostic assessment will assist the physicians in recognizing the high-risk patient situations, take timely interventions, and avoid delays or interruptions in treatment in cases of a high likelihood of a favorable net benefit.

Our current study has certain limitations. First, the nomogram is based on data from a single Chinese institution and cannot represent patients undergoing spinal internal fixation surgery in the whole country. Second, the risk factor analysis may not cover all potential factors contributing to the failure of spinal internal fixation surgery. Third, although the robustness of our nomogram was validated by the validation test, no external validation was performed, and a wider range of external assessments for patients undergoing spinal instrumentation surgery is required.

Although the current study has some limitations, our study provides a novel way to predict the risk of instrumentation failure after spinal internal fixation surgery. We identified four features to predict the risk of instrumentation failure in the spine. The selected features can be prioritized for extensive validation to demonstrate their clinical value.

## 5. Conclusion

The current research has developed a novel nomogram with moderate accuracy that can help clinicians in assessing the risk of instrumentation failure in patients undergoing earlier spinal instrumentation procedures, thereby providing early necessary intervention and reasonable postoperative guidance. However, this nomogram requires extensive external validation to determine whether interventions for risk factors can effectively reduce instrumentation failure in patients undergoing spinal instrumentation surgery.

## Figures and Tables

**Table 1:** Differences in characteristics between normal and failure groups of spinal instrumentation devices in the training test.

Demographic characteristics	Normal group	Failure group	Overall
	(*N* = 744)	(*N* = 55)	(*N* = 799)
Gender			
Female	370 (49.7%)	33 (60.0%)	403 (50.4%)
Male	374 (50.3%)	22 (40.0%)	396 (49.6%)
Age			
< =45	211 (28.4%)	32 (58.2%)	243 (30.4%)
>45	533 (71.6%)	23 (41.8%)	556 (69.6%)
BMI			
<18.5	75 (10.1%)	16 (29.1%)	91 (11.4%)
18.5-24.9	461 (62.0%)	28 (50.9%)	489 (61.2%)
25-29.9	184 (24.7%)	9 (16.4%)	193 (24.2%)
> =30	24 (3.2%)	2 (3.6%)	26 (3.3%)
Diagnosis			
Scoliosis	26 (3.5%)	16 (29.1%)	42 (5.3%)
Spinal fracture	77 (10.3%)	6 (10.9%)	83 (10.4%)
Spinal tumors	107 (14.4%)	4 (7.3%)	111 (13.9%)
Spinal tuberculosis	41 (5.5%)	4 (7.3%)	45 (5.6%)
Spinal degeneration	493 (66.3%)	25 (45.5%)	518 (64.8%)
Scoliosis			
No	718 (96.5%)	39 (70.9%)	757 (94.7%)
Yes	26 (3.5%)	16 (29.1%)	42 (5.3%)
Number of screws/number of vertebral bodies spanned			
<2	254 (34.1%)	30 (54.5%)	284 (35.5%)
2	486 (65.3%)	21 (38.2%)	507 (63.5%)
>2	4 (0.5%)	4 (7.3%)	8 (1.0%)
Number of screws/actual number of fixed vertebrae			
<2	115 (15.5%)	19 (34.5%)	134 (16.8%)
2	622 (83.6%)	32 (58.2%)	654 (81.9%)
>2	7 (0.9%)	4 (7.3%)	11 (1.4%)
Crosslink			
No	382 (51.3%)	32 (58.2%)	414 (51.8%)
Yes	362 (48.7%)	23 (41.8%)	385 (48.2%)
Unilateral or bilateral fixation			
Unilateral	58 (7.8%)	14 (25.5%)	72 (9.0%)
Bilateral	686 (92.2%)	41 (74.5%)	727 (91.0%)
Occupation			
Farmer	404 (54.3%)	28 (50.9%)	432 (54.1%)
Retired personnel	80 (10.8%)	5 (9.1%)	85 (10.6%)
Student	31 (4.2%)	10 (18.2%)	41 (5.1%)
Worker	23 (3.1%)	1 (1.8%)	24 (3.0%)
Other	206 (27.7%)	11 (20.0%)	217 (27.2%)
Marital status			
Unmarried	75 (10.1%)	16 (29.1%)	91 (11.4%)
Married	640 (86.0%)	38 (69.1%)	678 (84.9%)
Other	29 (3.9%)	1 (1.8%)	30 (3.8%)
Coronary heart disease			
No	731 (98.3%)	54 (98.2%)	785 (98.2%)
Yes	13 (1.7%)	1 (1.8%)	14 (1.8%)
Diabetes			
No	692 (93.0%)	53 (96.4%)	745 (93.2%)
Yes	52 (7.0%)	2 (3.6%)	54 (6.8%)
Hypertension			
No	616 (82.8%)	50 (90.9%)	666 (83.4%)
Yes	128 (17.2%)	5 (9.1%)	133 (16.6%)

**Table 2:** Differences in characteristics between normal and failure groups of spinal instrumentation devices in the validation test.

Demographic characteristics	Normal group	Failure group	Overall
	(*N* = 306)	(*N* = 34)	(*N* = 340)
Gender			
Female	147 (48.0%)	15 (44.1%)	162 (47.6%)
Male	159 (52.0%)	19 (55.9%)	178 (52.4%)
Age			
< =45	85 (27.8%)	23 (67.6%)	108 (31.8%)
>45	221 (72.2%)	11 (32.4%)	232 (68.2%)
BMI			
<18.5	26 (8.5%)	6 (17.6%)	32 (9.4%)
18.5-24.9	194 (63.4%)	20 (58.8%)	214 (62.9%)
25-29.9	74 (24.2%)	8 (23.5%)	82 (24.1%)
> =30	12 (3.9%)	0 (0%)	12 (3.5%)
Diagnosis			
Scoliosis	15 (4.9%)	9 (26.5%)	24 (7.1%)
Spinal fracture	30 (9.8%)	3 (8.8%)	33 (9.7%)
Spinal tumors	40 (13.1%)	2 (5.9%)	42 (12.4%)
Spinal tuberculosis	16 (5.2%)	6 (17.6%)	22 (6.5%)
Spinal degeneration	205 (67.0%)	14 (41.2%)	219 (64.4%)
Scoliosis			
No	293 (95.8%)	25 (73.5%)	318 (93.5%)
Yes	13 (4.2%)	9 (26.5%)	22 (6.5%)
Number of screws/number of vertebral bodies spanned			
<2	101 (33.0%)	22 (64.7%)	123 (36.2%)
2	199 (65.0%)	11 (32.4%)	210 (61.8%)
>2	6 (2.0%)	1 (2.9%)	7 (2.1%)
Number of screws/actual number of fixed vertebrae			
<2	39 (12.7%)	15 (44.1%)	54 (15.9%)
2	261 (85.3%)	17 (50.0%)	278 (81.8%)
>2	6 (2.0%)	2 (5.9%)	8 (2.4%)
Crosslink			
No	160 (52.3%)	18 (52.9%)	178 (52.4%)
Yes	146 (47.7%)	16 (47.1%)	162 (47.6%)
Unilateral or bilateral fixation			
Unilateral	15 (4.9%)	6 (17.6%)	21 (6.2%)
Bilateral	291 (95.1%)	28 (82.4%)	319 (93.8%)
Occupation			
Farmer	167 (54.6%)	17 (50.0%)	184 (54.1%)
Retired personnel	38 (12.4%)	3 (8.8%)	41 (12.1%)
Student	13 (4.2%)	5 (14.7%)	18 (5.3%)
Worker	7 (2.3%)	4 (11.8%)	11 (3.2%)
Other	81 (26.5%)	5 (14.7%)	86 (25.3%)
Marital status			
Unmarried	33 (10.8%)	9 (26.5%)	42 (12.4%)
Married	263 (85.9%)	25 (73.5%)	288 (84.7%)
Other	10 (3.3%)	0 (0%)	10 (2.9%)
Coronary heart disease			
No	301 (98.4%)	34 (100%)	335 (98.5%)
Yes	5 (1.6%)	0 (0%)	5 (1.5%)
Diabetes			
No	288 (94.1%)	34 (100%)	322 (94.7%)
Yes	18 (5.9%)	0 (0%)	18 (5.3%)
Hypertension			
No	257 (84.0%)	31 (91.2%)	288 (84.7%)
Yes	49 (16.0%)	3 (8.8%)	52 (15.3%)

**Table 3:** Prediction factors for spinal internal fixation device failure.

Intercept and variable	Prediction model				
	*β*	Odds ratio	2.5% CI	97.5% CI	*P* value
Intercept	-1.035	0.355	0.156	0.764	0.010
Gender	-0.438	0.645	0.351	1.167	0.151
Age	-0.797	0.451	0.240	0.852	0.013
Scoliosis	1.988	7.301	3.290	16.013	<0.001
Unilateral or bilateral fixation	-1.363	0.256	0.126	0.543	<0.001

*β* is the regression coefficient; CI, confidence interval.

## Data Availability

The datasets used and/or analyzed during the present study are available from the corresponding author on reasonable request.

## Abbreviations

LASSO: Least absolute shrinkage and selection operator
CI: Confidence interval
AUC: Area under the curve
SE: Standard error.
